# Intelligent Algorithm-Based Gastrointestinal X-Ray Examination in Evaluating the Therapeutic Effect of Probiotics Combined with Triple Therapy on Children with *Helicobacter* Infection

**DOI:** 10.1155/2022/8464361

**Published:** 2022-03-27

**Authors:** Qizheng Wang, Jiangshu Li

**Affiliations:** Department of Pediatrics, Huai'an Maternal and Child Health Care Hospital, Huai'an 223002, Jiangsu, China

## Abstract

In this study, gastrointestinal X-ray imaging was processed based on the Ncut algorithm, the gastric signs of the children after probiotics combined with triple therapy were examined, the therapeutic effect of probiotics combined with triple therapy was evaluated, and the correlation between *Helicobacter pylori* (HP) infection and gastric disease was analyzed. The included children were randomly divided into group A (treated with standard triple therapy) and group B (pretreated with probiotics on the basis of the treatment method of group A) with 48 cases in each group. The gastrointestinal angiography results of children were observed. The accuracy of the gastrointestinal angiography based on intelligent algorithms was evaluated by taking the results of the urea breath test (UBT) as the gold standard. The results were as follows: first, gastrointestinal X-ray imaging before and after treatment showed that the recovery of the gastric body and gastric antral mucosa for children in group B was better than that of group A (*P* < 0.05); second, the incidence of gastrointestinal diseases in HP-positive patients was 78% and the incidence of HP-negative patients was 32%; third, the sensitivity, specificity, and accuracy of gastrointestinal X-ray imaging based on intelligent algorithm were 76.47%, 93.67%, and 90.63%, respectively. After treatment, tumor necrosis factor *α* (TNF-*α*) and interleukin 6 (IL-6) in group B were much lower than those in group A (*P* < 0.05), and the incidence of adverse reactions in group B was lower than that in group A (*P* < 0.05). In summary, gastrointestinal X-ray imaging based on intelligent algorithm had a reliable reference value for the judgment of gastrointestinal HP infection and probiotics combined with triple therapy was more effective for HP infection, which was worthy of clinical application.

## 1. Introduction

*Helicobacter pylori* (HP) infection is a global matter affecting the health of patients, and it increases with age, seriously affecting the health of children [1]. At present, the detection methods of HP infection mainly include gastroscopy and gastric mucosal pathological examination. Most scholars advocate active clinical treatment after childhood HP infection. In recent years, intestinal microecology studies have found that HP maintains close contact with gastric mucosal epithelial cells, so methods to correct the imbalance of gastrointestinal flora may be effective in the treatment and prevention of HP infection [2–4]. Traditional triple therapy has a certain degree of drug resistance due to the extensive use of drugs in clinical practice, and the effect is poor [5–7]. Studies have found that probiotics can release bacteriocins or organic acids and inhibit the growth of HP by destroying the cell wall or cytoplasmic membrane [8, 9]. Probiotics can also compete with HP to bind glycolipid receptors and adhere to gastric epithelial cells to reduce their mucosal damage to gastric epithelial cells [10]. In addition, probiotics can stabilize the gastric mucosal barrier, reduce inflammation, exert antioxidant effects, and promote the healing of damaged mucosa [11]. Endoscopic biopsy is also one of the most commonly used tests for HP infection. In addition, specific detection methods include rapid urease testing and silver-stained biopsies. However, there are few studies on X-ray imaging of the stomach and duodenum in patients with HP infection. Gastrointestinal X-ray combined with gas-barium double contrast filling compression method was adopted, and the patient's gastrointestinal tract was inflated by using a gas generating agent. Then, the patient is given a dry suspension of barium sulfate, and the patient's position is adjusted so that it can evenly apply the barium solution on the surface of the stomach cavity. Different body positions can be filmed under gas-assisted conditions to avoid omission of lesions. This study was applied to improve gastrointestinal X-ray imaging for the detection of HP-infected gastropathy.

In this work, the gastrointestinal X-ray imaging based on the Ncut algorithm was applied to evaluate the clinical efficacy of probiotics combined with triple therapy in the treatment of children with HP infection. Using the urea breath test (UBT) results as the gold standard, the accuracy of the test results and the relationship between HP infection and gastrointestinal X-ray imaging in children were evaluated. By detecting intestinal microecology and inflammatory factors, the effect of compound *Lactobacillus acidophilus* tablets was analyzed, the effects of different treatment methods on TNF-*α* and IL-6 in children were explored, and the incidence of adverse reactions of children with different treatment methods was analyzed. It aimed to provide treatment options for diseases caused by HP infection.

## 2. Research Methods

### 2.1. Research Objects

A total of 96 children with HP infection who were hospitalized from October 2019 to October 2020 were selected as the research objects. The children had upper gastrointestinal symptoms such as epigastric pain, discomfort, and anorexia. The children included were randomly divided into group A and group B, with 48 cases in each group. The children in group A were 3–12 years old, with an average age of 6.47 ± 2.78, while the children in group B were 3–11 years old, with an average age of 6.59 ± 2.13 years. The radiological approach did not pose a risk of radiation accumulation to the children. The study had been approved by the Ethics Committee of hospital, and the families of patients and children were informed about the study and signed informed consent.

The inclusion criteria were as follows: children whose 13C breath test was positive but with no proton pump inhibitor (PPI) within 2 weeks and no history of use of antibacterial drugs and bismuth within one month; children whose parents had signed the informed consent forms.

The exclusion criteria were as follows: children with severe liver, kidney, and cardiac dysfunction; children with mental system diseases; children with previous digestive system surgery; and children with history of allergy to the drugs in this study or similar drugs.

### 2.2. Grouping and Dosage Regimens of Children

Children in group A received standard triple therapy treatment: omeprazole with 0.6–1.0 mg/kg/d, amoxicillin with 50 mg/kg/d (maximum dose 1.0 g, 2 times/d), and clarithromycin with 15–20 mg/kg/d (maximum dose 0.5 g, 2 times/d). All of the above medicines were taken before breakfast and dinner with 10 days in total.

Children in group B received the following dosage regime. Before the triple therapy, the compound *Lactobacillus acidophilus* tablets (1 tablet/time, 0.5 g/tablet) were taken with cold boiled water after three meals for 2 weeks. Next, triple therapy (same as group A) was taken for 2 weeks. After completion, the compound *Lactobacillus acidophilus* tablets (1 tablet/time, 0.5 g/tablet) were taken again for 2 weeks.

### 2.3. Gastrointestinal X-Ray Imaging Based on Intelligent Algorithm

The noise gray value in the X-ray image was obviously different from its surrounding normal gray value, so the domain averaging algorithm in the spatial denoising algorithm was used to remove the noise in the image. First, it was assumed that the actual gray value in the image was *f(u, v)*, and the gray value of adjacent pixels can be expressed as *M*_*i*_(*i*=1,2,…, *n*); then, it can be expressed as the following equation by using the domain average method:

(1) $$\begin{array}{c} g\left(u,v\right)=\left\{\begin{array}{c} \frac{1}{n}\overset{n}{\underset{i=1}{\sum }}M_{i},\left|f\left(u,v\right)-\frac{1}{n}\overset{n}{\underset{i=1}{\sum }}M_{i}\right|>\sigma ,\\ f\left(u,v\right),\text{ other.} \end{array}\right. \end{array}$$

In the above equation, *s* refers to the error threshold, which is a constant.

It was further assumed that the domain average was a low-pass filter of the image *f(u, v)*, and the discrete convolution was adopted for noise removal in the image. After the average value of the image was calculated, the image with noise smoothing function was finally obtained, which can be expressed as follows:

(2) $$\begin{array}{c} g\left(x,y\right)=\frac{1}{n}\overset{n}{\underset{k=1}{\sum }}{\left[f\left(x,y\right)\right]}_{k}. \end{array}$$

To improve the effect of X-ray image denoising and enhancement, curvelet transform was adopted to process the image. Basic nonlinear enhancement operators included GAG operator, and then new operators can be proposed:

(3) $$\begin{array}{c} f\left(x,\mu \right)=\left\{\begin{array}{c} \frac{x-c\mu }{c\mu }{\left(\frac{m}{c\mu }\right)}^{\alpha }+\frac{2c\mu -x}{c\mu },\;x<2c\mu ,\\ {\left(\frac{m}{x}\right)}^{\alpha },\;2c\mu \leq x<m,\\ {\left(\frac{m}{x}\right)}^{\beta },\;x\geq m. \end{array}\right. \end{array}$$

In the above equation, *μ* is the noise standard deviation, *α* is the determinant of the degree of nonlinear transformation, *β* refers to the determinant of the dynamic compression range, *c* represents the regularization parameter, and *m* is the threshold.

*µ* in (3) could be estimated as the following equation based on the wavelet analysis method:

(4) $$\begin{array}{c} \mu =\frac{\left[abs{\left(C\left\{a\right\}\left\{b\right\}\right)}_{\text{median}}\right]}{0.67}. \end{array}$$

In equation (4), C is the subband coefficient of the image, *a* refers to the scale, *b* is the direction, and *abs*(*C*{*a*}{*b*})_median_ represents the median of the absolute value of the subband coefficient.

Then, the Ncut algorithm was applied to segment the target area in the X-ray image. The similarity matrix of the traditional Ncut algorithm can be written as follows:

(5) $$\begin{array}{c} Z_{uv}=e^{\left(-F_{u}-{F_{v}}_{2}^{2}/\sigma_{l}^{2}\right)}\left\{\begin{array}{c} e^{\left(-x_{u}-{x_{v}}_{2}^{2}/\sigma_{x}^{2}\right)},\;x\left(u\right)-x{\left(v\right)}_{2}>r,\\ 0,\text{ other.} \end{array}\right. \end{array}$$

In equation (5), *F*_*u*_ is the gray value of the pixel point *u*, *x(u)* is the spatial position of the pixel point *u*, and *σ*_*l*_ and *σ*_*x*_ are the sensitivities of the gray level and position, respectively.

**Figure 1:**
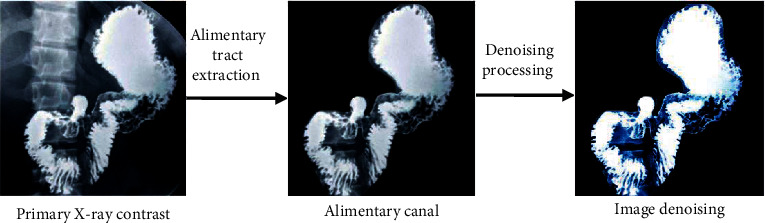
The processing process of gastrointestinal X-ray imaging using intelligent algorithm.

The specific image processing performance is shown in Figure 1.

### 2.4. Examinations

All patients received gastrointestinal X-ray angiography. In addition, all patients were required to take oral barium sulfate contrast agent before the examination. The time required for the entire inspection was about 10–15 minutes. First, a chest X-ray examination was performed to observe whether the heart and lungs had pathological changes, if there were foreign bodies that are not transparent to X-rays, and if there were contraindications such as obstruction or perforation. Second, the passage of the contrast agent in the esophagus was observed to examine whether there was ulcer of the esophagus and esophageal cancer. Third, the contrast agent passing through the cardia again was observed to check if there was achalasia and cardia cancer. After that, the patient was required to lay on the examination table and quickly turned 1 to 2 circles from right to left to observe the fundus of the stomach, the gastric antrum mucosa, and the gastric filling image. Finally, the patient was required to stand and scan from the esophagus to the stomach again. Then, the intelligent X-ray image segmentation method was adopted to process the obtained X-ray images.

The 13C breath test was performed, and HP routine examination required stopping antibacterial drugs for at least 4 weeks, stopping proton pump inhibitors for at least 2 weeks, and fasting on the day of the test. The 13C UBT delta over baseline (DOB) was used as an indicator, and DOB value > 4.4 was positive and DOB value < 3.6 was negative. They need to be taken orally with warm water completely, and do not crush them, so as not to affect the accuracy of the test results.

The intestinal microecological test was conducted as follows. Before and after treatment, 2 g of fresh fecal specimens were collected from all patients, which were diluted by the 10-fold dilution method, inoculated on the surface of the culture medium plate, and then evenly spread with *L* glass rods for routine aerobic and anaerobic culture. The most representative aerobic bacteria (*Enterococcus*, *Enterobacter*, and yeast) and anaerobic bacteria (*Bifidobacterium*, *Lactobacillus*, *Clostridium perfringens*, and *Bacteroides*) in the intestinal flora were selected for cultivation and bacterial identification using French bioMérieux identification system. The result was calculated as the logarithm (lgCFU/g) of the number of colonies (CFU) per gram of wet stool weight, the intestinal colonization resistance (B/E ratio) was calculated, and the B/E value was calculated by calculating the ratio of bifidobacteria and enterobacteria (B/E value) represented intestinal colonization resistance.

5 mL of fasting peripheral venous blood was collected from the two groups of patients before and after treatment and centrifuged at 3000 r/min for 10 minutes using the centrifuge to separate the serum. The enzyme-linked immunosorbent assay (ELISA) was applied to detect the serum tumor necrosis factor *α* (TNF-*α*) and interleukin 6 (IL-6) levels.

### 2.5. Observation Indicators

The gastrointestinal X-ray imaging results of the two groups of patients were compared before and after treatment, mainly to observe the thickening of the gastric body and gastric mucosal folds. The specific standards are shown in Table 1.

The 13C UBT DOB of patients between the two groups was compared before and after treatment. The judgment standard was as follows: DOB value > 4.4 was positive, and DOB value < 3.6 was negative. The negative test result was expressed as HP eradicate, and the occurrence of nausea and vomiting, diarrhea, abdominal distension, abdominal pain, rash, and other adverse reactions in the two groups of patients was recorded.

### 2.6. Statistical Analysis

The SPSS 22.0 was applied for statistical analysis. The measurement data were expressed as $\bar{x}\pm s$, the counting data were expressed as percentages, and the independent sample *t*-test and *χ*^2^ test were applied, respectively. *P* < 0.05 indicated that the difference was statistically significant.

## 3. Results

### 3.1. Basic Data

In group A and group B, there were 48 cases in each group, and there was no statistically obvious difference in gender, age, average course of disease, and disease composition between the two groups, as shown in Table 2.

### 3.2. 13C Breath Test Results

**Figure 2:**
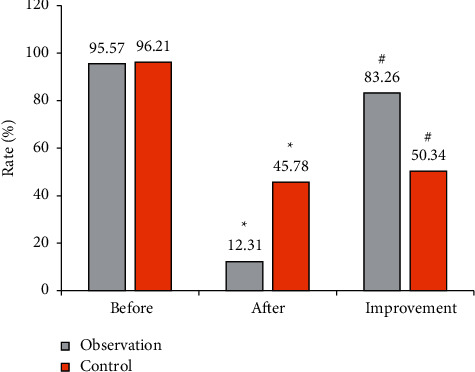
Comparison on 13C breath test results. ^∗^Comparison of positive rates between the two groups after treatment, *P* < 0.05. ^#^Comparison of improvement rates between the two groups, *P* < 0.05.

Figure 2 shows the results of the 13C breath test of the two groups before and after treatment. Before treatment, the positive rates of HP infection in group B and group A were 95.57% and 96.21%, respectively, while they were 12.31% and 45.78%, respectively, after the treatment. After analysis and comparison, the improvement rate of group B HP infection (83.26%) was greatly higher than that of group A (50.34%), and the difference was statistically great (*P* < 0.05).

### 3.3. Comparison on HP Removal Rate

The HP clearance rate of group A and group B was 52.08% and 89.58%, respectively, so the therapeutic effect of group B was much higher than that of group A (*P* < 0.05), as shown in Table 3.

### 3.4. Changes of Gastrointestinal X-Ray Imaging

**Figure 3:**
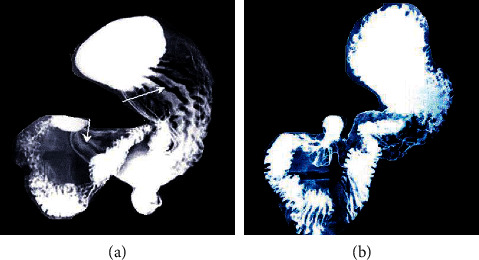
Results of gastrointestinal X-ray imaging. (a) Normal X-ray of gastrointestinal tract. (b) Positive gastrointestinal changes in HP.

Figure 3(a) is a normal X-ray image of the stomach, and the position indicated by the white arrow in Figure 3(a) refers to the folded part of the gastric mucosa. As shown, the outline of the stomach was smooth and tidy, the greater curvature of the stomach body was jagged, and the folds of the gastric mucosa can be seen in three obvious vertical and horizontal oblique shapes. The body of the stomach was parallel, the smaller curvature of the stomach was neat, and the stomach and greater curvature were grid-like. In addition, obvious vertical, horizontal, and oblique lines were also visible in the antrum. Figure 3(b) shows the HP-positive gastrointestinal changes in this study: stomach body and antral mucosal folds were thickened, disordered, and blurred, and gastric antrum folds were circular.

### 3.5. Gastrointestinal X-Ray Pattern in the Diagnosis of HP Infection

**Figure 4:**
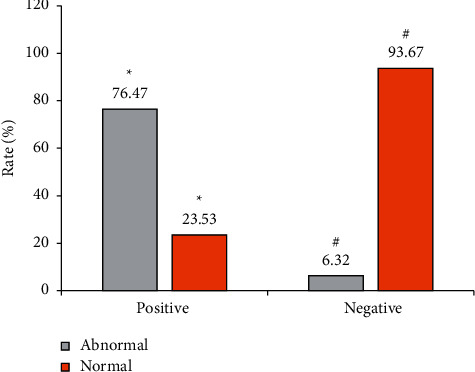
Comparison of HP results and X-ray imaging results. ^∗^Comparison of positive rates between the two groups after treatment, *P* < 0.05. ^#^Comparison of improvement rates between the two groups, *P* < 0.05.

Table 4 shows the statistical results between the performance of gastrointestinal X-ray imaging after treatment and the HP-positive rate. After analysis and comparison, it was found that the probability of abnormal X-ray signs of HP-positive patients was 76.47% and that of those without abnormality was 23.53%, showing no statistical difference (*P* < 0.05). The probability of abnormalities in X-ray signs of HP-negative patients was 6.32%, and that of those without abnormalities was 93.67%, showing statistically great difference (*P* < 0.05), as shown in Figure 4. According to Table 4, the sensitivity, specificity, and accuracy of the X-ray examination results were 76.47%, 93.67%, and 90.63%, respectively.

### 3.6. Comparison of Changes in Intestinal Flora and Inflammatory Factors

Before treatment, there was no observable difference in the distribution of intestinal flora between the two groups of patients (*P* > 0.05). Compared with the condition before treatment, patients in group A showed a significant decrease in *Enterobacter*, yeast, *Clostridium perfringens*, *Bifidobacterium*, and *Lactobacillus* after treatment (*P* < 0.05) and a significant increase in enterobacteria (*P* < 0.05), and the B/E ratio was greatly reduced (*P* < 0.05). After treatment in group B patients, bifidobacteria and lactobacilli were remarkably increased (*t* = 2.468 and 3.887, respectively, *P* < 0.05), *Clostridium perfringens* was greatly reduced (*P* < 0.05), enterococci and yeasts were reduced observably (*P* < 0.05), enterobacteria did not change observably (*P* > 0.05), and the B/E value was increased markedly (*P* < 0.05). The results revealed that the B/E value of group B patients after treatment was much higher than that of group A (*P* < 0.05). As shown in Table 5, there was no significant difference in TNF-*α* and IL-6 between the two groups of children before the treatment (*P* > 0.05), but those in group B were both greatly lower than group A after the treatment, showing statistically observable differences (*P* < 0.05) (Table 6).

### 3.7. Occurrence of Adverse Reactions

There were 3 cases of nausea and vomiting, 2 cases of diarrhea, 3 cases of abdominal distension and abdominal pain, 2 cases of loss of appetite, and 4 cases of rash in group A. There were 1 case of nausea and vomiting and 1 case of rash in group B. Therefore, the incidence of adverse reactions in group B was lower than that of group A (*P* < 0.05) (as given in Table 7).

## 4. Discussion

Under normal circumstances, the beneficial and harmful bacteria in the human gut are in a balanced state to maintain human health. When this balance is disrupted, the distribution of gut flora changes and the gut flora becomes unbalanced, which may lead to various diseases [12]. *Lactobacillus* and *Bifidobacterium* are beneficial bacteria in the human gut, while *Enterobacter* and *Enterococcus* are opportunistic pathogens in the human gut [13, 14]. Some studies have found that supplementary probiotic triple therapy has good efficacy and safety in eradicating HP in children. Fang et al. [15] conducted a meta-analysis of randomized controlled trials to supplement the efficacy of lactic acid bacteria triple therapy in the treatment of HP infected children, and the results showed that lactic acid bacteria as an adjunctive triple therapy can improve the eradication rate of HP and reduce the incidence of treatment-related diarrhea in children. Studies have shown that compound *Lactobacillus acidophilus* can promote the recovery of gastric mucosal permeability, protect the integrity of the mucosa, further prevent HP invasion, and form a mucosal barrier *in vivo* [16, 17]. The compound *Lactobacillus acidophilus* can reduce the production of inflammatory factors such as interleukin and interferon in the process of Th1 cell response, induce inflammatory factors such as interleukin produced by Th2 cell response, reduce mucosal inflammation, and enhance resistance to HP [18]. TNF-*α* and IL-6 are common inflammatory factors, and their blood levels show corresponding changes when inflammation occurs, and increases in its levels can be seen in various infectious diseases and have significant signs of illness in HP-infected patients [19, 20].

Among the medical image segmentation algorithms, the FCM algorithm has the advantages of being easy to execute, convenient, and fast and has become one of the mainstream segmentation algorithms [21]. The Ncut algorithm can promote the development of machine learning algorithms and is worthy of clinical application and promotion. Applying the Ncut algorithm to the medical field can help doctors solve some image processing tasks and help reduce the workload of doctors. The combination of Ncut algorithm and medical image processing is a combination of mathematics and medicine, which is beneficial to the progress of medical technology. Matsumoto et al. [22] used an intelligent algorithm to diagnose heart failure using chest X-ray images, and the results showed a diagnostic accuracy of 82%, suggesting that intelligent algorithms can help support the use of chest X-ray images to diagnose heart failure.

In this work, the gastrointestinal X-ray imaging based on intelligent algorithm was used to examine the digestive tract and the results of 13C breath test were used as the standard. The results of the 13C breath test showed that the improvement rates of the positive results of HP before and after treatment in group B and group A were 83.26% and 50.34%, respectively. Therefore, the improvement rate of group B was significantly higher than that of group A, indicating that the use of probiotics combined with triple therapy is superior to pure triple therapy. The sensitivity, specificity, and accuracy of X-ray examination results were 76.47%, 93.67%, and 90.63%, respectively, indicating that gastrointestinal X-ray imaging is feasible in the detection of HP infection. It was found that the serum levels of TNF-*α* and IL-6 in patients in group B were significantly lower than those in patients in group A, indicating that probiotics combined with triple therapy can significantly improve the intestinal flora of children with HP infection than triple therapy alone. In addition, the incidence of adverse reactions in group B was lower than that in group A (*P* < 0.05).

## 5. Conclusion

The intelligent X-ray image segmentation method had good effect in gastrointestinal X-ray image processing. Probiotics combined with triple therapy had a good clinical effect on children with *Helicobacter pylori* infection. It can better improve the clearance rate of *Helicobacter pylori*, improve the inflammatory response, and effectively improve the intestinal microecology of patients. The effect was remarkable, and it was worthy of clinical application.

## Figures and Tables

**Table 1:** Thickening criteria for gastric mucosal fold.

Position	Specific value
Antrum of stomach	≥0.5 cm
Gastric body	≥0.7 cm

**Table 2:** Basic data of patients in two groups.

	Group A	Group B
Age (years old)	7.38 ± 2.84	7.42 ± 2.69
Gender (male/female)	25/23	24/24
Average course of disease	5.38 ± 1.51	5.36 ± 1.49
Chronic gastritis	20	22
Gastric ulcer	11	11
Duodenal ulcer	17	15

**Table 3:** Comparison on HP removal rate in patients of two groups.

Group	Group A	Group B
HP removal rate (%)	52.08	89.58^#^

^#^The difference was statistically obvious in contrast to group A (*P* < 0.05).

**Table 4:** Gastrointestinal X-ray pattern in the diagnosis of HP infection.

Gastrointestinal X-ray signs	13C breath test results		Total number of cases
	Positive	Negative	
Number of children with abnormal results	13	5	18
Number of children with normal results	4	74	78
Total number	17	79	96

**Table 5:** Changes of intestinal flora before and after treatment in the two groups (lgCFU/g).

Group	Group A		Group B	
	Before treatment	After treatment	Before treatment	After treatment
*Enterobacter*	7.97 ± 0.72	8.64 ± 0.72^∗^	7.96 ± 0.69	8.03 ± 0.75#
*Enterococcus*	6.48 ± 0.65	6.31 ± 0.75	6.50 ± 0.67	6.10 ± 0.85^∗^#
Yeast	5.10 ± 0.67	4.84 ± 0.76^∗^	5.06 ± 0.67	4.33 ± 0.74^∗^#
*Bifidobacterium*	7.42 ± 0.63	6.96 ± 0.62^∗^	7.41 ± 0.70	7.98 ± 0.81^∗^#
*Lactobacillus*	6.47 ± 0.57	5.77 ± 0.61^∗^	6.42 ± 0.63	6.68 ± 0.63^∗^#
*Clostridium perfringens*	5.90 ± 0.42	5.41 ± 0.43^∗^	5.87 ± 0.51	5.13 ± 0.47^∗^#
B/E value	0.93 ± 0.12	0.79 ± 0.11^∗^	0.93 ± 0.11	0.99 ± 0.12^∗^#

^∗^ *P* < 0.05 in contrast to the value before treatment in the same group; ^#^*P* < 0.05 in contrast to the value in the same period between two different groups.

**Table 6:** Comparison of cytokines between the two groups of children.

Group	Group A		Group B	
	Before treatment	After treatment	Before treatment	After treatment
TNF-*α* (ng/L)	452.42 ± 43.28	397.42 ± 54.74^∗^	462.46 ± 49.54	225.85 ± 61.47^∗^#
IL-6 (ng/L)	59.74 ± 5.15	43.08 ± 10.33^∗^	60.19 ± 4.80	32.42 ± 6.13^∗^#

^∗^ *P* < 0.05 in contrast to the value before treatment in the same group; ^#^*P* < 0.05 in contrast to the value in the same period between two different groups.

**Table 7:** Comparison on occurrence of adverse reactions of children.

Group	Group A	Group B
Nausea and vomiting	3	1
Diarrhea	2	0
Bloating/abdominal pain	2	0
Loss of appetite	2	0
Rash	4	1
Total	13	2#

^#^ *P* < 0.05 in contrast to group A.

## Data Availability

The data used to support the findings of this study are available from the corresponding author upon request.
